# Enhancement of Thermal Diffusivity in Phase-Separated Bismaleimide/Poly(ether imide) Composite Films Containing Needle-Shaped ZnO Particles

**DOI:** 10.3390/polym9070263

**Published:** 2017-07-02

**Authors:** Shoya Uchida, Ryohei Ishige, Shinji Ando

**Affiliations:** Department of Chemistry and Material Engineering, Tokyo Institute of Technology, Ookayama, Meguro-ku, Tokyo 152-8552, Japan; excellented@hotmail.co.jp (S.U.); rishige@polymer.titech.ac.jp (R.I.)

**Keywords:** polymer blend, thermal diffusivity, soluble polyimide, bismaleimide, needle-shaped zinc oxide

## Abstract

Phase-separated polymer blend composite films exhibiting high thermal diffusivity were prepared by blending a soluble polyimide (BPADA-MPD) and a bismaleimide (BMI) with needle-shaped zinc oxide (n-ZnO) particles followed by high-temperature curing at 250 °C. Images recorded with a field-emission scanning electron microscope (FE-SEM) equipped with wavelength-dispersive spectroscopy (WDS) demonstrated that the spontaneously separated phases in the composite films were aligned along the out-of-plane direction, and the n-ZnO particles were selectively incorporated into the BMI phase. The out-of-plane thermal diffusivity of the composite films was significantly higher than those of the previously reported composite films at lower filler contents. Based on wide-angle X-ray diffraction (WAXD) patterns and image analysis, the enhanced thermal diffusivity was attributed to the confinement of the anisotropically shaped particles and their nearly isotropic orientation in one phase of the composite films.

## 1. Introduction

The minimization and advanced performance of state-of-the-art electronic devices urgently require improvements in heat dissipation from electronic components loaded on flexible printed circuit boards. Since heat is transported along the out-of-plane direction in insulating dielectric layers, the out-of-plane thermal conductivity of polymer films adhering to these components is particularly important. Although the thermal conductivity of polymer films is significantly lower than that of metallic and inorganic materials, an effective way to enhance the thermal conductivity of polymeric films is by generating a highly ordered structure such as crystalline or liquid-crystalline-like phases. Controlling the orientation of liquid crystalline polymers (LCPs) using a magnetic field has been reported to effectively enhance the thermal conductivity [1]. However, a strong external field must be applied to the LCP films for a long time to align the polymer chains.

**Figure 1:**
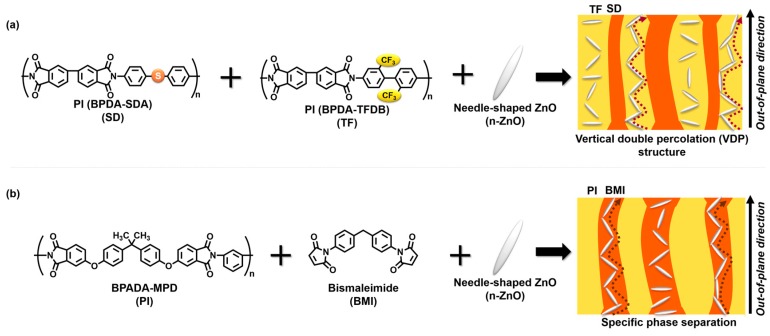
Specific morphology formed from phase separation of an immiscible (**a**) PI blend; and a (**b**) BMI/BPADA-MPD blend with needle-shaped ZnO (n-ZnO) particles. The red/brown arrows and the white ellipsoids represent directions of thermal conduction and n-ZnO particles, respectively.

Incorporating inorganic particles (fillers) with high thermal conductivity into a polymer matrix is another way to enhance the thermal conductivity of polymers. In general, when these inorganic fillers are homogeneously dispersed in a polymer matrix, the thermal conductivity of the composite is scarcely enhanced, due to the imperfect formation of percolation pathways due to insufficient contact between the fillers. Thus, using non-spherical, anisotropically shaped inorganic fillers is a judicious way to enhance the thermal conductivity of composite films. Compared with spherical fillers, anisotropic fillers have a higher probability of making contact with each other due to their high aspect ratios [2]. In a previous report, we demonstrated that the degree of orientation of anisotropic, platelet-shaped fillers in a composite film is nearly proportional to the anisotropy of thermal conductivity of composite films [2], controlling the orientation of anisotropic fillers is key to enhancing the out-of-plane thermal conductivity. Recently, we succeeded in preparing novel composite polyimide (PI) films containing anisotropic fillers [3]. As shown in Figure 1, a blend film was prepared by mixing poly(amic acid)s (PAAs) of BPDA-TFDB (TF) and BPDA-SDA (SD) with needle-shaped ZnO (n-ZnO) particles, followed by thermal curing at 350 °C for 1.5 h under a nitrogen atmosphere. A phase-separated structure spontaneously formed in the blend composite films, due to the poor miscibility between the fluorine-containing PAA and the sulfur-containing PAA. In addition, the two phases of PAAs were separately aligned along the out-of-plane direction during drying and thermal curing, which was designated a vertical double percolation (VDP) structure [4,5]. Since more n-ZnO particles were selectively incorporated into the TF phase (matrix phase) of the blend composite film, the orientation of the fillers was randomized by the confinement effect, and the nearly isotropic orientation of the n-ZnO particles significantly enhanced the thermal conductivity in the blend composite films. Hence, our previous study revealed that generating a phase-separated structure effectively increases the out-of-plane thermal conductivity of the composite materials.

On the other hand, a characteristics phase separation morphology was reported for a blend material derived from a soluble BPADA-MPD PI and a cross-linkable bismaleimide (BMI) resin [6,7]. According to the reference [7], the sizes of the island and sea phases could be controlled by adjusting the ratio of the amounts of components. Using this blend system, the fillers could be further confined, and the anisotropic fillers could be partly aligned along the out-of-plane direction of the composite films. In this study, we attempted to prepare new types of polymer blend composite films composed of a soluble PI (BPADA-MPD), BMI, and n-ZnO particles (Figure 1). Moreover, the relationship between the out-of-plane thermal diffusivity of the composite films and their phase-separated morphology was investigated using analytical methods such as scanning electron microscopy (SEM) and wide-angle X-ray diffraction (WAXD).

## 2. Experimental Section

### 2.1. Materials

4,4′-(4,4′-Isopropylidenediphenoxy)bis(phthalic anhydride) (BPADA), kindly supplied by Manac Co. Ltd. (Hiroshima, Japan), was used as received. 4,4′-Methylenedianiline (MPD) and 4,4′-thiodianiline (SDA) were purchased from Wako Co. Ltd. (Tokyo, Japan) and sublimated under reduced pressure before use. Bismaleimide (BMI) was purchased from Tokyo Chemical Industry Co. Ltd. (Tokyo, Japan), *N*,*N*-dimethylacetamide (DMAc, anhydrous) was purchased from Sigma-Aldrich (St. Louis, MO, USA), and both were used as received. Needle-shaped zinc oxide (n-ZnO) particles were synthesized according to the literature [8], and the experimental details and the properties of the particles have been reported elsewhere [3]. FE-SEM micrographs of aggregated and individual n-ZnO particles are shown in Figure S1 (Supporting Information). The length and diameter of the needle-shaped crystallites were 2–5 μm and 100–300 nm, respectively. In addition, the monodispersity of n-ZnO particles after dispersion treatment by an ultrasonic homogenizer was monitored by particle size analyser (PSA). The PSA profiles before and after the treatment are shown in Figure S2. The monomodal dispersion observed after treatment is attributable to the size distribution of mono-dispersed n-ZnO particles (0.3–7 μm). The zeta potential of n-ZnO particles dispersed in ethanol was estimated as 26.3 ± 3 mV, which agrees well with the reference values of ZnO particles (24.0, 26.3 and 26.8 mV at pH 7) [9,10]. This positive and large zeta potential could facilitate homogenous dispersion of n-ZnO particles in polyimide and cured BMI matrices.

BMI resin films containing 0–25 vol % of n-ZnO particles were prepared according to the following procedure. Firstly, n-ZnO particles were dispersed in DMAc using a planetary centrifugal mixer (Thinky ARE-310, Thinky Co., Tokyo, Japan), and BMI was added to the dispersion. Then, the solution was casted onto a glass substrate and thermally cured at 155 and 250 °C for 1 h each under a nitrogen atmosphere.

Two kinds of PAA solutions were prepared by mixing (a) equimolar amounts of BPADA and MPD; and (b) BPADA, MPD and SDA at a molar ratio of 50:37.5:12.5 in NMP followed by stirring for one day. The solid contents of BPADA-MPD and BPADA-MPD/BPADA-SDA were preset to 15 and 17 wt %, respectively. The PAA solutions containing 0–26 vol % of n-ZnO particles were prepared according to literature [3,5]. The PAA solutions were casted onto glass substrates and thermally cured at 250 °C for 1 h.

An organic-solvent-soluble PI solution containing n-ZnO was prepared by stirring the mixed solution of n-ZnO particles dispersed in NMP and PI (BPADA-MPD) film dissolved in DMAc or NMP for 18 h. To prepare the mixture solution of BPADA-MPD and BMI, BMI was added to the BPADA-MPD solution and stirred for 4 h, in which the weight ratios of BPADA-MPD and BMI were set to 25:75. The solutions were spin-coated onto the glass substrate and thermally cured by the same procedure as BMI. The film thickness was controlled to ca. 40–45 μm.

### 2.2. Measurements

Zeta (ζ) potential of n-ZnO particles dispersed in ethanol was measured with a laser zeta potentiometer ELS-8000 (Otsuka Electronics Co., Osaka, Japan). The value was obtained with the Smoluchowski equation by using the electrophoretic mobility, which was corrected for the electro-osmosis effect. Each measurement was repeated 5 times at a scattering angle a 20°. Surface images of BMI/PI/n-ZnO composite films were taken with an Olympus SZX12 microscope (Olympus Co., Tokyo, Japan). Cross-sectional images of the films were observed by desktop SEM (TM3000, Hitachi, Tokyo, Japan) using the backscattering electron method. WAXD patterns were obtained using a Bruker D8 DISCOVER (Bruker, Billerica, MA, USA) with Cu-K_α_ radiation (50 kV, 100 mA) equipped with a Vantec 500 detector. The detector was aimed such that the normal to the detector plane was tilted by 40° from the X-ray irradiation direction to detect the diffraction from n-ZnO in the wide-angle region. Fourier transform infrared (FT-IR) spectra were recorded with a Nicolet Avatar FT-IR 320 spectroscopy (Thermo Fisher Scientific, Waltham, MA, USA) using the attenuated total reflection (ATR) method or the KBr method. The thermal expansion behaviors of PI films (BPADA-MPD, BPADA-SDA, and copolymers) were evaluated by Shimadzu TMA-60 (Shimadzu, Kyoto, Japan) with a fixed load of 1.5 g and a heating rate of 10 °C/min. Differential scanning calorimetry (DSC, Shimadzu DSC-60, Shimadzu, Kyoto, Japan) was used to monitor the cross-linking reaction of BMI. The temperature was raised from room temperature to 300 °C at a heating rate of 10 °C/min. The thermal diffusivities along the out-of-plane direction (*D*_⏊_) of PI and BMI/PI composite films were measured at room temperature using an AC temperature wave analyzer (ai-Phase mobile 1u, ai-phase Co. Ltd., Tokyo, Japan) [11,12]. Each film was measured ten times at different locations, and the average value was adopted as the experimental value. An elemental analysis of the composite films was performed by wavelength-dispersive spectroscopy (WDS) equipped with field-emission SEM (FE-SEM, JXA-8200, JEOL, Akishima, Tokyo, Japan). Image J software developed by the National Institutes of Health was used to analyze the area fraction of each phase of the composites, wherein the Tsai method [13] was adopted for setting the thresholds for binarization.

## 3. Results and Discussion

### 3.1. Characterization of BMI and BPADA-MPD

**Figure 2:**
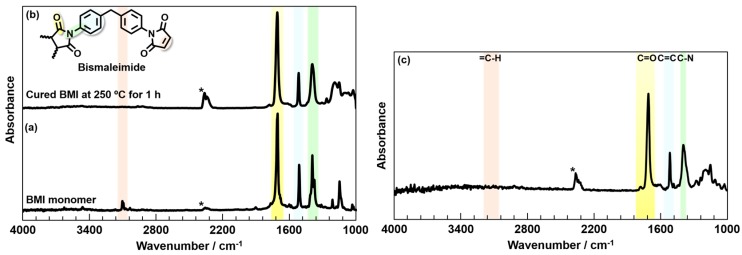
ATR-FT-IR spectra of (**a**) BMI monomer; (**b**) cured BMI resin; and (**c**) BMI/BPADA-MPD blend films. The BMI resin and BMI/PI blend film were cured at 250 °C for 1 h.

The polyimide (PI) and BMI/PI blend composite films were characterized using DSC, attenuated total reflection (ATR)-FT-IR, and a thermal mechanical analysis (TMA). BMI has been reported to exhibit an exothermic peak at 207 °C in DSC, which was attributed to its cross-linking reactions [14]. The DSC curves of the BMI monomer and a BMI resin thermally cured at 250 °C for 1 h are presented in Figure S3. The endothermic peak appearing at 162 °C and the broad exotherm starting around 165 °C and peaking around 207 °C are assignable to the melting of BMI crystallites and the cross-linking reactions, respectively. In contrast, these peaks do not appear for the cured BMI resin. Similarly, in the FT-IR spectra, the stretching vibration band attributable to the =C–H double bond in the monomer at 3100 cm^−1^ disappeared with the progress of cross-linking [14]. The FT-IR spectra of both the BMI monomer and resin (Figure 2a,b) clearly exhibit the C=O asymmetric stretching band (1720 cm^−1^) and C–N stretching band (1380 cm^−1^) derived from the imide ring and C=C stretching band (1490 cm^−1^) from the benzene rings. Meanwhile, the =C–H stretching band of the maleimide structure (3100 cm^−1^) was only observed for the BMI monomer, indicating that the cross-linking was completed by curing at 250 °C for 1 h. In addition, the BMI/PI blend cured at 250 °C was characterized by FT-IR (Figure 2c). Since the =C–H stretching vibration band of maleimide was not observed, and the C–N stretching and C=O symmetric and asymmetric vibrations bands of imide structure were confirmed, the cross-linking reaction of BMI and the thermal imidization of BPADA-MPD were completed after curing at 250 °C.

**Figure 3:**
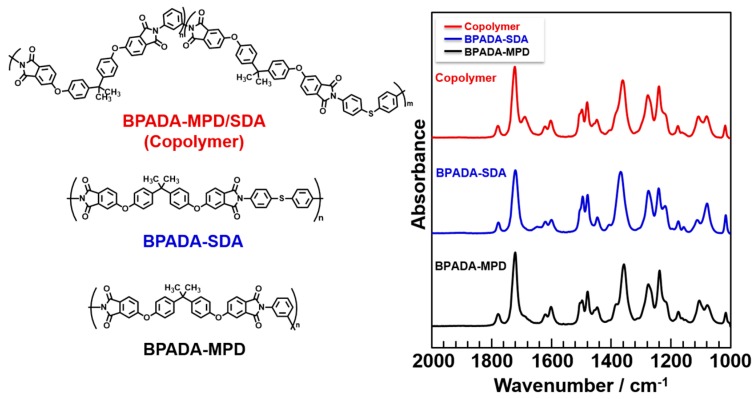
Structures and ATR-FT-IR spectra of PI (BPADA-SDA/MPD, BPADA-SDA, and BPADA-MPD) films.

The ATR-FT-IR spectra for the three kinds of PIs (BPADA-MPD, BPADA-SDA/MPD, and BPADA-SDA) cured at 250 °C are presented in Figure 3. All spectra clearly demonstrated the C=O asymmetric (1780 cm^−1^) and C=O symmetric (1720 cm^−1^) stretching bands assignable to the imide ring and the C–N (1380 cm^−1^) stretching band assignable to the imide-phenyl linkage. The TMA curves of the PI films are summarized in Figure S4 and Table S1. The glass transition temperature (*T*_g_: 229 °C) and the coefficient of thermal expansion (CTE: 47–56 ppm/K), which were estimated by TMA for BPADA-MPD, are in good agreement with the results of a previous study [15]. The PI films of BPADA-SDA and BPADA-MPD/SDA showed relatively lower *T*_g_ values (220 and 200 °C) because of their flexible thioether linkages in the main chains. The *T*_g_ values of all PIs are lower than 250 °C, and thus, the thermal imidization should be completed after curing at 250 °C for 1 h.

### 3.2. Characterization and Morphology of BMI/PI Composite Films

**Figure 4:**
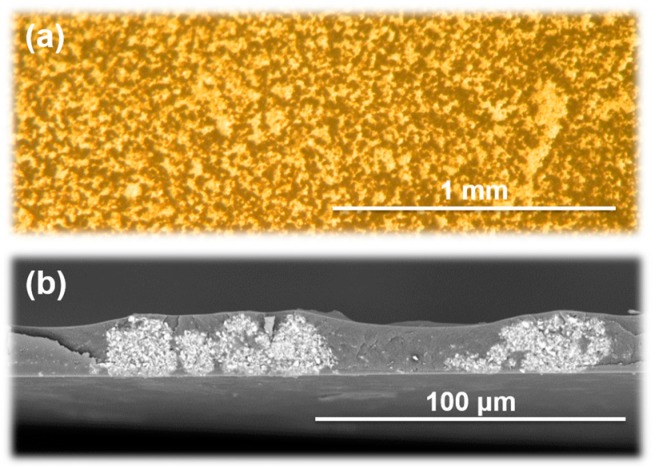
(**a**) Optical microscopic photographs; and (**b**) cross-sectional SEM image of BMI/BPADA-MPD blend films containing n-ZnO particles (7 vol %).

BMI/BPADA-MPD composite films were prepared by thermally curing precursor solutions containing n-ZnO particles at 155 °C and 250 °C for 1 h each. Figure 4 presents photographs of the surface of a composite film containing 7 vol % of n-ZnO captured by an optical microscope and a cross-sectional SEM image. Dark and bright regions are clearly separated in the surface image, which indicates that distinct phase separation occurred in the blend film, wherein the introduced n-ZnO particles were preferentially incorporated into one of these phases. The SEM image also shows bright and dark regions representing the phase-separated structure, which are aligned along the out-of-plane direction. Therefore, the surface images and SEM micrographs clearly confirmed that the VDP structure [4,5] was successfully formed in the blend composite film.

**Figure 5:**
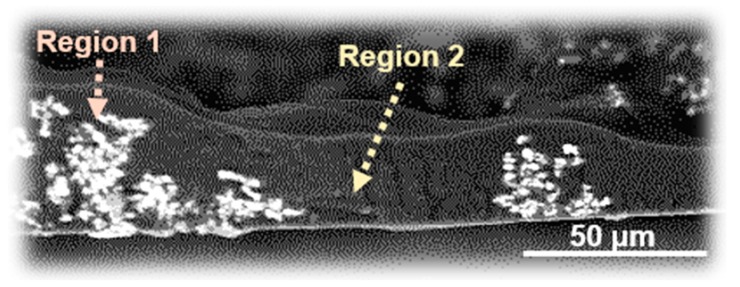
Cross-sectional SEM image of the BMI/copolyimide blend films containing n-ZnO particles (5 vol %).

In order to characterize the components of the island and sea phases in the BMI/PI blend composite films, an elemental analysis was performed by SEM-WDS. Since no heavy atoms are contained in the both matrices, BPADA-MPD was slightly modified by copolymerization with a sulfur-containing SDA diamine (MPD: SDA = 75:25 mol %). The cross-sectional SEM image shown in Figure 5 demonstrates that two phases are separately aligned along the out-of-plane direction, wherein the phase separation was driven by the segregation of BPADA-MPD and cured BMI. The results of the WDS analysis for the phases with and without n-ZnO particles are listed in Table 1. The ZnO-rich and ZnO-poor phases in Figure 5 correspond to Region 1 and Region 2, respectively. Clearly, the sulfur content in Region 2 is significantly higher than that of Region 1. This difference indicates that the ZnO-poor phase was mainly composed of the copolyimide-containing SDA, which confirms that the ZnO-rich and ZnO-poor phases correspond to the BMI-rich and PI-rich phases, respectively.

The higher affinity of the n-ZnO particles to BMI than to PIs can be supported by estimating the interfacial energies of the components as follows. The wetting coefficient ω is useful for estimating the affinity between zinc oxide and each polymer phase, and it is calculated by the following equation [16].

(1) $$\omega \;=\;\frac{\Gamma_{\mathrm{PI}-\mathrm{ZnO}}-\Gamma_{\mathrm{BMI}-\mathrm{ZnO}}}{\Gamma_{\mathrm{PI}-\mathrm{BMI}}}$$

where Γ_PI-BMI_ is the interfacial energy between BPADA-MPD and BMI, and Γ_PI-ZnO_ and Γ_BMI-__ZnO_ are those between ZnO particles and each polymer. When ω > 1, ZnO particles will be preferentially incorporated into the BMI-rich phase, while in the case of ω < −1, ZnO particles will be mainly located in the BPADA-MPD-rich phase. On the other hand, when −1 < ω < 1, ZnO particles could be located at the interface between the BPADA-MPD-rich and BMI-rich phases. The interfacial energy Γ_1–2_ between two components (1 and 2) is calculated as follows [17,18].

(2) $$\Gamma_{1-2}\;={\;\Gamma }_{1}+{\;\Gamma }_{2}-2\left(\sqrt{\Gamma_{1}^{d}\Gamma_{2}^{d}}+\sqrt{\Gamma_{1}^{p}\Gamma_{2}^{p}}\right)$$

where Γ_1_ and Γ_2_ are the surface energies of the respective components, and the superscripts *d* and *p* denote the dispersive and the polar components of the surface energy, respectively. The surface energies of the three components are listed in Table 2 [19,20,21]. Consequently, the wetting coefficient of the polymer blend system containing n-ZnO particles is estimated to be 3.59, which predicts that n-ZnO particles will be preferentially incorporated into the BMI-rich phase. This analysis is obviously in good agreement with the SEM-WDS results.

### 3.3. Out-of-Plane Thermal Diffusivities

**Figure 6:**
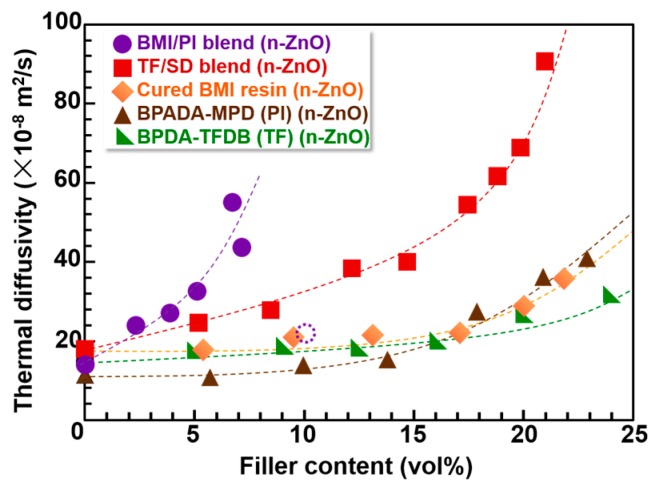
Filler-content dependence of the out-of-plane thermal diffusivities (*D*_⏊_) for various PI, PI blend, and BMI/PI blend composite films. The significantly reduced *D*_⏊_ value observed for the BMI/PI blend film with a filler content of 10 vol % is denoted by a dotted circle.

The out-of-plane thermal diffusivity (*D*_⏊_) of the PI, BMI, and BMI/PI blend composite films evaluated by TWA at room temperature are summarized in Figure 6 and Table S2. For comparison, the *D*_⏊_ values of the previously reported PI blend composite films (TF/SD) and homopolyimide (TF) films containing n-ZnO particles are also presented [3]. Firstly, the *D*_⏊_ values of BMI composite films (orange diamonds) are slightly higher than those of BPDA-TFDB (TF) composite films (green triangles). This could be due to three-dimensional cross-linking structures and the absence of the in-plane orientation of the cured BMI matrix. In the BMI composite film, no self-standing blend films were obtained for n-ZnO contents higher than 30 vol % due to the high rigidity and brittleness of the films. Secondly, in the case of BPADA-MPD composite films, the *D*_⏊_ values (brown triangles) are slightly lower than those of the TF composite films at lower loadings (<15 vol %). The inherent *D*_⏊_ value of pristine BPADA-MPD film is lower than that of pristine TF film because of the flexible ether and bent *m*-phenylene linkages in its main chain, which coincides well with the analysis proposed in our previous study [22]. In contrast, the *D*_⏊_ of the TF composite film was lower than that of BPADA-MPD composite film in the higher loading region. We previously reported that when PIs that prefer isotropic orientation are used as matrices, the orientation of the anisotropic fillers became more isotropic in the composite films [23,24]. Since the BPADA-MPD chains are more randomly oriented than that of TF, n-ZnO particles were more isotropically oriented in the BPADA-MPD composite films. Finally, TF/SD blend composite films showed significantly larger *D*_⏊_ values than those of homopolymer composite films, PI and TF, owing to the enhanced confinement effect and isotropic orientation of n-ZnO.

On the other hand, the BMI/PI blend films containing n-ZnO particles presented significantly larger *D*_⏊_ values than all the other composite films containing immiscible PI blend films composed of TF and SD PIs. We recently reported that n-ZnO particles incorporated into PI blend films are more randomly oriented than those in pristine PI films, and the percolation threshold was significantly lowered by the reduced in-plane orientation of anisotropic fillers [3]. The above result strongly suggests that n-ZnO particles are more isotropically oriented in the BMI/BPADA-MPD blend films.

### 3.4. Image Analysis of Optical Microscope Photographs

**Figure 7:**
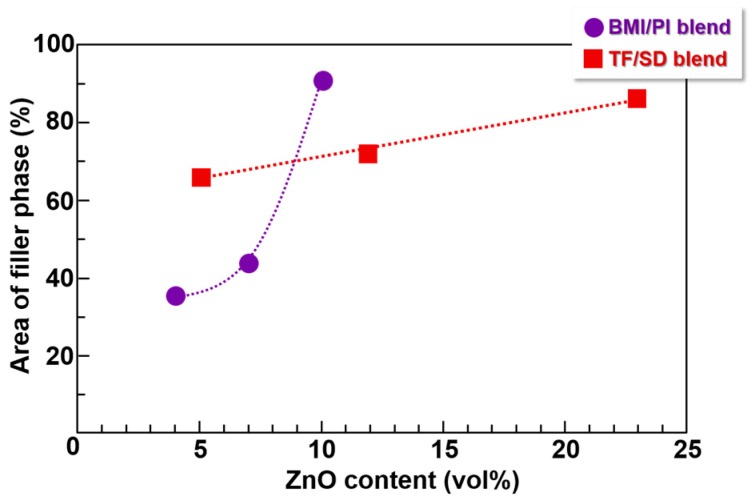
Variation in the area containing n-ZnO particles with the ZnO content for BMI/PI and TF/SD blend films.

To estimate the area fractions of ZnO-rich phase in the BMI/PI and TF/SD blend composite films, a built-in function of the Image J software was used to analyze optical microscope images of these films, such as Figure 4a in the present study and Figure 3c in ref. [3]. To quantitatively estimate the surface area fraction of ZnO-rich phase, the “area fraction” function was applied to the images after the binarization of each pixel. Then, the percentage of black pixels relative to the total number of pixels was defined as the fraction of the area containing n-ZnO particles. As summarized in Figure 7, the area fraction containing n-ZnO particles in the VDP structures of TF/SD blend films was proportional to the filler content, suggesting that the fraction of the volume containing n-ZnO particles increases with the filler content. In contrast, the area fraction of n-ZnO rich phase in the BMI/PI composite films at small loadings (<7 vol %) is remarkably lower, which indicates that the fillers are well confined in the phase-separated BMI phase of the VDP structure, as shown in Figure 4b. However, the fraction steeply increased with an increase in the filler content. In the BMI/PI composite films containing 10 vol % n-ZnO, the fillers are nearly homogeneously dispersed, as shown in Figure S5, and the VDP structure completely collapsed. In the blend composite system, the viscosities of polymer matrix solutions should be carefully adjusted because the solution viscosity significantly increased with the increasing filler content. In the case of the TF/SD blend solution [3,5], the viscosity of each PI solution with or without fillers could be separately adjusted. However, the viscosity of BMI monomer solutions containing fillers was hardly adjusted in this study. Therefore, the solution viscosity of the blend system simultaneously increased with the increasing filler content. This could explain the drastic change in the morphology, that is, the collapse of VDP structure, in the BMI/PI composite films with the higher filler contents. This morphological change caused a steep decrease in *D*_⏊_ from 54.1 × 10^−8^ m^2^/s (filler content: 7 vol %) to 20.5 × 10^−8^ m^2^/s (10 vol %) in Figure 6, which also demonstrates that the VDP structure is a very effective way to form thermally conductive pathways along the out-of-plane direction.

### 3.5. Orientation Analysis of Composite Films by WAXD

**Figure 8:**
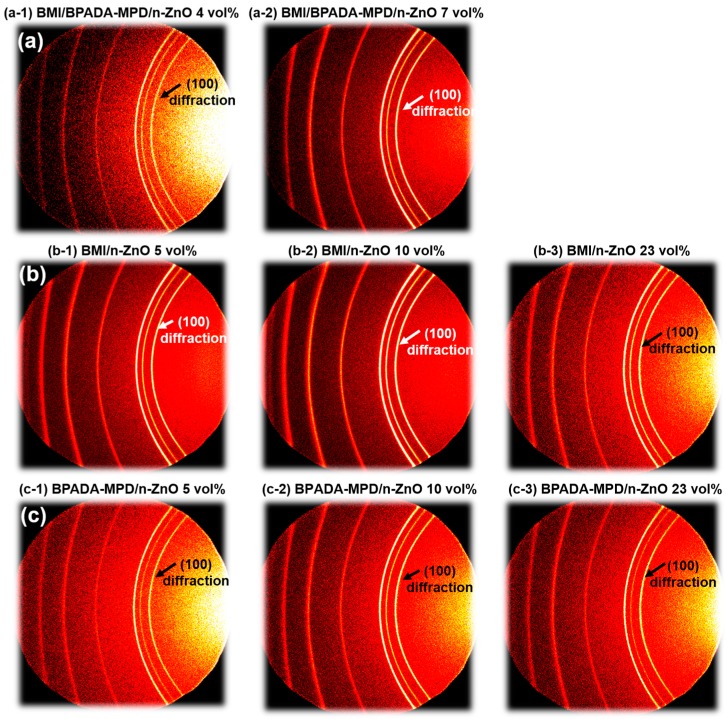
WAXD patterns of n-ZnO particles incorporated into (**a**) BMI/BPADA-MPD/n-ZnO blend (4 and 7 vol %); (**b**) BMI/n-ZnO (5, 10 and 23 vol %); and (**c**) BPADA-MPD/n-ZnO (5, 10 and 23 vol %) composite films.

**Figure 9:**
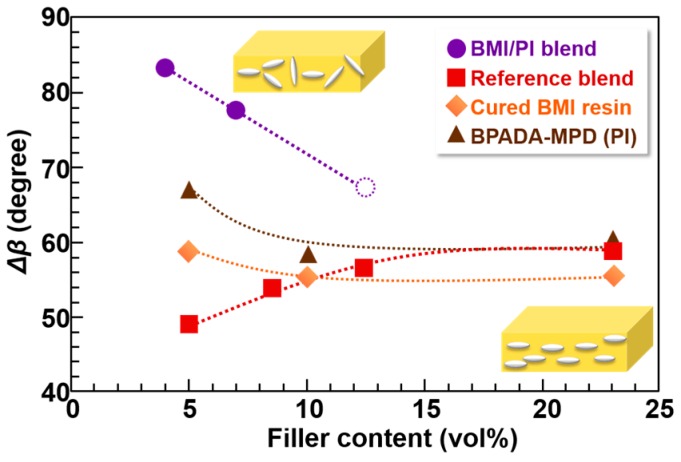
Full-width at half-maximum of the azimuthal intensity distribution of (100) diffraction, Δ*β*, for four kinds of composite films with various n-ZnO contents.

As mentioned in the Introduction, controlling the orientation of anisotropic fillers and the phase-separation morphology are crucial to enhancing the out-of-plane thermal conductivity in composite films. To examine the orientations of the n-ZnO particles in the composite films, wide-angle X-ray diffraction (WAXD) measurements were performed. Since the diffraction patterns of n-ZnO are observed in the wide-angle region beyond 2θ = 30° [25], thereby exceeding the CCD detection area of the diffractometer, the normal to the detector plane was tilted 40° from the direction of the X-ray radiation to detect very wide-angle diffraction [3]. The WAXD patterns of n-ZnO particles incorporated into the composite films are shown in Figure 8. The diffractions of the (100) and (002) planes for n-ZnO were observed in the patterns [26]. Hence, the orientation of the dispersed n-ZnO particles in the matrix can be analyzed by the azimuthal intensity distribution of the (100) direction. Figure 9 presents the variations in the full-width at half-maximum, Δβ, of the intensity distribution in the azimuthal direction for the (100) diffractions of n-ZnO. When the n-ZnO particles are oriented in the in-plane direction, the distribution of the (100) diffraction intensity as a function of β leads to a small Δβ. In contrast, when the fillers are more randomly oriented, the (100) diffraction intensity distribution becomes quite broad, with a large Δβ. The Δβ values of n-ZnO particles observed for the TF/SD PI blend composite films are also plotted for comparison [3]. Notably, the Δβ values of the BMI and BPADA-MPD composite films are significantly higher than those of TF/SD blend composite films at lower filler loadings (<7 vol %). This indicates that the out-of-plane component of the orientation vector of n-ZnO particles incorporated into the BMI/PI blend films is larger than those in the TF/SD films. As previously described [3], when PI blend composite films were prepared with n-ZnO particles, the in-plane orientation of n-ZnO particles were effectively obstructed by boundaries between two phases, which induced the relatively isotropic orientation of the n-ZnO particles. Additionally, the volume fraction of the ZnO-rich phase in the BMI/PI blend composite films was smaller than that in the TF/SD PI blend composite films at low filler loadings (Figure 7). Therefore, the n-ZnO particles were more isotropically oriented in the BMI/PI composite films owing to the reduced volume fraction of the ZnO-rich phase and the boundaries between BMI and PI phases. This structural character is beneficial to preparing composite materials exhibiting higher thermal conductivity with very low filler contents and to lowering the percolation threshold of the composite materials. However, the Δβ values for the azimuthal intensity distribution of n-ZnO particles in the BMI/PI, PI, and BMI composite films were gradually decreased with the increase in the filler content, except for the TF/SD composite films. The narrowing of the (100) diffraction peaks of the azimuthal intensity profile indicates that as the content of the anisotropic n-ZnO particles increased, the particles gradually oriented to the in-plane direction in the matrices [3], which coincides with the increase in the area fraction of ZnO-rich phase (Figure 7). In contrast, the out-of-plane orientation of the n-ZnO particles in the TF/SD composite films gradually increased with the aid of the VDP structures [3]. These results confirm that promoting the alignment of the n-ZnO particles along the out-of-plane direction by controlling the morphology of the phase-separated structures is a very effective way to enhance the *D*_⏊_ of the blend composite materials.

## 4. Conclusions

The out-of-plane thermal diffusivity (*D*_⏊_) of novel BMI/polyimide (BPADA-MPD) blend composite films containing needle-shaped ZnO particles was investigated based on structural and morphological analyses using ATR-FT-IR, FE-SEM with WDS, WAXD, and image analysis. The ZnO-rich and ZnO-poor phases were separately aligned along the out-of-plane direction in the composite films, exhibiting the VDP structure, and the anisotropic fillers were selectively incorporated into the BMI-rich phase due to the difference in wettability between the BMI and BPADA-MPD matrices. At lower filler contents (<7 vol %), the *D*_⏊_ values of the novel composite films were significantly higher than those of the previously reported composite films. A surface image analysis clarified that the area fraction of the ZnO-rich phase in the novel composite films was significantly smaller than those of the previously prepared composite films, and thus, the n-ZnO particles were more isotropically oriented in the blend composite films. However, the *D*_⏊_ values significantly decreased, accompanied by a steep increase in the area fraction of the ZnO-rich phase due to the collapse of the VDP structure at higher filler contents (>10 vol %). Consequently, the significant increase in *D*_⏊_ observed in the novel blend composite films could be attributed to the effective formation of thermally conductive pathways along the out-of-plane direction due to the closer confinement and the randomized orientation of the n-ZnO particles in the composite films.

## Figures and Tables

**Table 1:** Elemental compositions analyzed by WDS in Regions 1 and 2 in Figure 5.

Region	Carbon (%)	Oxygen (%)	Sulfur (%)	Zinc (%)
1	58.85	13.29	0.09	4.01
2	61.25	13.84	0.53	0.33

**Table 2:** Surface energies of BPADA-MPD, BMI resin, and ZnO powder [19,20,21].

Materials	Surface Energy (mJ/m^2^)	Dispersive Component (mJ/m^2^)	Polar Component (mJ/m^2^)
BPADA-MPD	35.1	32.9	2.2
BMI	31.3	25.0	6.3
ZnO powder	40.5	22.0	18.5

## Supplementary Materials

The following are available online at www.mdpi.com/2073-4360/9/7/263/s1, Figure S1: FE-SEM image of aggregated n-ZnO and needle-shaped n-ZnO particles; Figure S2: Particle size dispersions of n-ZnO (a) before and (b) after dispersion treatment measured by a particle size analyzer (Microtrac HRA9320, Honeywell International Inc., Morris Plains, NJ, USA); Figure S3: DSC thermograms of BMI monomer and BMI resin prepared by thermally curing at 250 °C for 1 h; Figure S4: TMA curves of PI (BPADA-SDA/MPD, BPADA-SDA, and BPADA-MPD) films; Figure S5: Cross-sectional SEM image of BMI/BPADA-MPD composite film (ZnO content: 10 vol %); Table S1: Thermomechanical data of PI films; Table S2: n-ZnO content (φ_p_) and thermal diffusivity *D*_⏊_ of PI (BPADA-MPD), BMI, and BMI/PI blend composite films. These data are plotted in Figure 6.
