# Supplementary material for: Enhancement of Thermal Diffusivity in Phase-Separated Bismaleimide/Poly(ether imide) Composite Films Containing Needle-Shaped ZnO Particles
Source: Polymers (Basel). 2017 Jul 2;9(7):263. doi: 10.3390/polym9070263 (PMC6432426; doi:10.3390/polym9070263)
Supplement: Supplementary file 1 [file polymers-09-00263-s001.pdf]

Supporting Information:

Enhancement of Thermal Diffusivity on Phase-separated Bismaleimide/Poly(ether imide) Composite Films Containing Anisotropic Fillers

Shoya UCHIDA, Ryohei ISHIGE and Shinji ANDO*

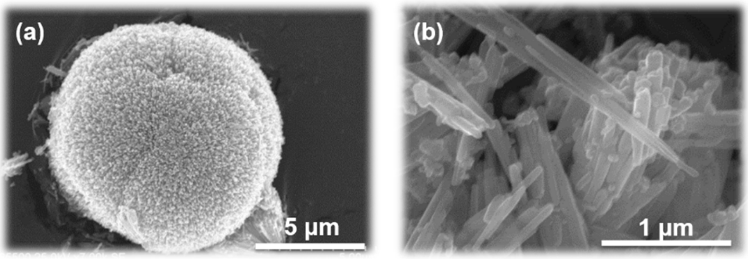

Figure S1. FE-SEM image of aggregated n-ZnO and needle-shaped n-ZnO particles.

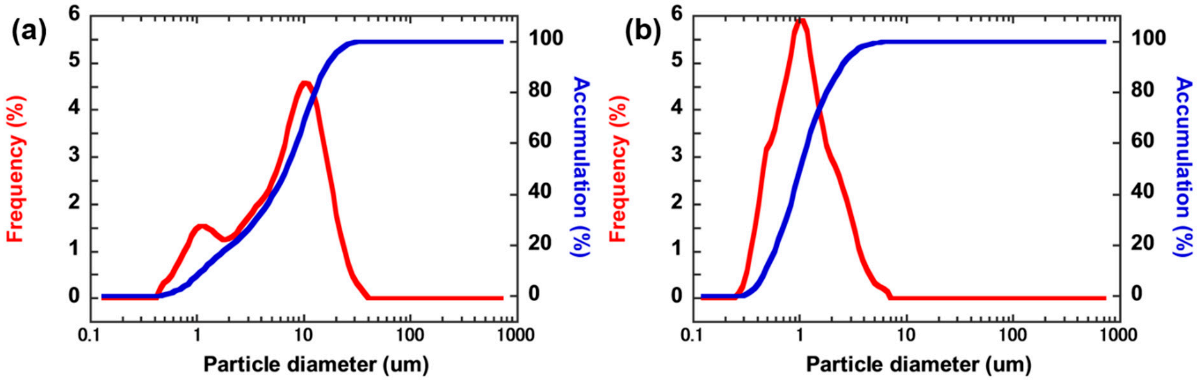

Figure S2. Particle size dispersions of n-ZnO (a) before and (b) after dispersion treatment measured by a particle size analyser (Microtrac HRA9320, Honeywell International Inc.).

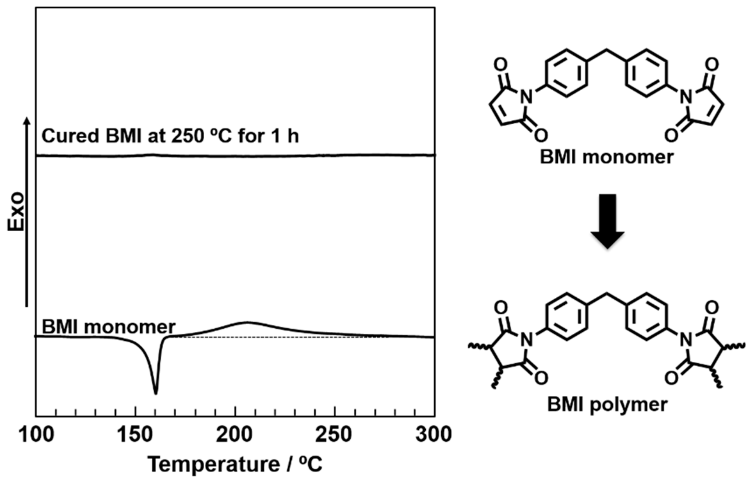

Figure S3. DSC thermograms of BMI monomer and BMI resin prepared by thermally curing at 250 °C for 1 h.

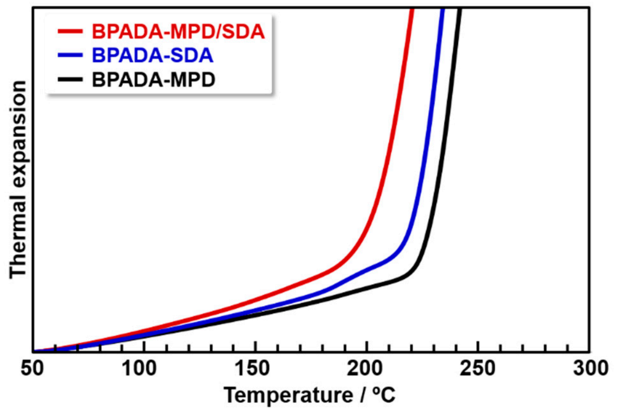

Figure S4. TMA curves of PI (BPADA-SDA/MPD, BPADA-SDA, and BPADA-MPD) films.

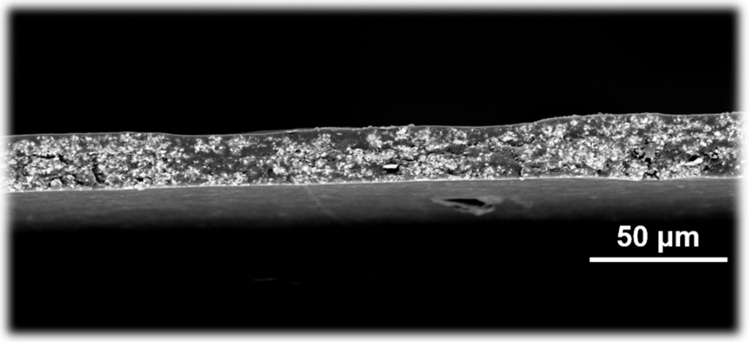

Figure S5. Cross-sectional SEM image of BMI/BPADA-MPD composite film (ZnO content: 10 vol%).

Table S1. Thermomechanical properties of PI films estimated from Figure S3.

Materials	T_g [a] [°C]	CTE [b] (80-130 °C) [ppm/K]	CTE (130-180 °C) [ppm/K]
BPADA-MPD	229	46.3	56.1
BPADA-SDA	220	52.9	66.1
BPADA-MPD/SDA	200	64.3	89.8

[a] Glass transition temperature, [b] Coefficient of linear thermal expansion.

Table S2. n-ZnO content (ϕ_P) and thermal diffusivity $D_\perp$ of PI (BPADA-MPD), BMI, and BMI/PI blend composite films. These data are plotted in Figure 6.

Composite films	ϕ_P [vol%]	Average $D_\perp$ [10^{-8} m^2/s]	Standard deviation
BPADA-MPD-0	0	11.4	1.2
BPADA-MPD-6	5.8	10.4	0.7
BPADA-MPD-10	10.0	13.1	1.9
BPADA-MPD-14	13.9	15.0	1.4
BPADA-MPD-18	18.0	26.3	2.4
BPADA-MPD-21	21.0	35.7	3.4
BPADA-MPD-23	23.0	39.7	3.3
BMI-0	0	12.6	0.6
BMI-5	5.4	17.3	1.9
BMI-10	9.5	20.2	1.8
BMI-13	13.3	21.6	1.9
BMI-17	17.2	22.1	2.7
BMI-20	20.2	29.0	2.3
BMI-22	22.0	35.6	8.2
BMI/PI blend-0	0	13.0	1.2
BMI/PI blend-3	2.5	23.9	8.0
BMI/PI blend-4	4.0	26.8	9.7
BMI/PI blend-6	5.5	32.3	16.9
BMI/PI blend-7	7.1	54.1	16.9
BMI/PI blend-8	7.5	43.8	17.1
BMI/PI blend-10	10.0	20.5	4.3
